# Impact of Drying Methods on Phenolic Composition and Bioactivity of Celery, Parsley, and Turmeric—Chemometric Approach

**DOI:** 10.3390/foods13213355

**Published:** 2024-10-23

**Authors:** Staniša Latinović, Ladislav Vasilišin, Lato Pezo, Nataša Lakić-Karalić, Dragoljub Cvetković, Aleksandra Ranitović, Sara Brunet, Teodora Cvanić, Jelena Vulić

**Affiliations:** 1 Faculty of Technology, University of Banja Luka, Bulevar Vojvode Stepe Stepanovića 73, 78000 Banja Luka, Bosnia and Herzegovina; stanisa.latinovic@tf.unibl.org (S.L.); ladislav.vasilisin@tf.unibl.org (L.V.); natasa.lakic-karalic@tf.unibl.org (N.L.-K.); 2 Institute of General and Physical Chemistry, University of Belgrade, Studentski Trg 12-16, 11000 Belgrade, Serbia; latopezo@yahoo.co.uk; 3 Faculty of Technology, University of Novi Sad, Bulevar cara Lazara 1, 21000 Novi Sad, Serbia; cveled@uns.ac.rs (D.C.); a.ranitovic@uns.ac.rs (A.R.); teodora.cvanic@uns.ac.rs (T.C.); 4 BioSense Institute, University of Novi Sad, Dr Zorana Đinđića 1, 21000 Novi Sad, Serbia; sara.brunet@biosense.rs

**Keywords:** celery, parsley, turmeric, drying, phenolics, antioxidant activity, statistical analysis, antihyperglycemic activity, antibacterial activity

## Abstract

Drying is one of the most commonly used methods for food preservation, and in spice processing, it has a significant impact on quality. In this paper, the influences of drying at room temperature, 60 °C, and 90 °C and freeze-drying on celery and parsley roots and turmeric rhizomes were examined. The highest content of total phenolics was found in celery dried at 60 °C (C60), parsley at room temperature (PRT), and freeze-dried turmeric (TFD) (1.44, 1.58, and 44.92 mg GAE/g_dm_, respectively). Celery dried at room temperature (CRT), PRT, and TFD showed the highest antioxidant activity regarding the DPPH and ABTS radicals and FRAP. The analysis of color parameters revealed that celery dried at 90 °C (C90); PFD and TFD showed the most similar values to control samples. The drying process was optimized using a combination of standard score (SS) and artificial neural network (ANN) methods. The ANN model effectively evaluated the significance of drying parameters, demonstrating high predictive accuracy for total phenolics, total flavonoids, total flavonols, total flavan-3-ols, IC_50_^ABTS^, and FRAP. TFD showed the strongest α-glucosidase inhibitory potential. Also, TFD extract showed good antibacterial activity against *Staphylococcus aureus* but not against *Escherichia coli*. C90 and PFD extracts did not show antibacterial activity against the tested microorganisms.

## 1. Introduction

It has been known since ancient times that spices carry out a significant role in the daily diet of a large portion of the human population. Due to their pleasant aroma, they are principally used during food preparation or added to ready-made meals to improve their sensory properties. It is, in addition, a fact that they exhibit numerous beneficial effects on human health, including antioxidant, antimicrobial, antihyperglycemic, and anticancer activities, and are useful in eliminating digestive and cardiovascular disorders [1,2,3]. That is why they are frequently used for therapeutic purposes. Thanks to their antioxidant and antimicrobial potential, spices prevent food spoilage and help preserve its freshness [4].

Celery (*Apium graveolens* L.) is a plant species that belongs to the Apiaceae family. All parts of this plant, including the leaves, stem, and root, are used in human nutrition. It is characterized by its high nutritional value and is an excellent source of antioxidants, minerals, fibers, and cellulose [5].

Parsley (*Petroselinum crispum* L.), like celery, belongs to the Apiaceae family. It is grown in tropical, subtropical, and temperate-continental regions. It is a biennial plant, but it is mostly grown as an annual. Parsley leaves and roots are used in human nutrition. Additionally, as a spice, parsley root is also known as a powerful diuretic [6].

Turmeric (*Curcuma longa* L.) is a spice plant that, according to plant systematics, belongs to the Zingiberaceae family. It is known to grow in tropical and subtropical areas, such as India, Pakistan, China, and Peru, and it is believed to be native to India. The rhizome of this plant is used in human nutrition and is an excellent source of polyphenolic compounds, flavonoids, and other antioxidants. Due to its chemical composition, it exhibits anti-inflammatory, antibacterial, antioxidant, and antiseptic effects in the human body [7,8]. Several studies have demonstrated the therapeutic potential of turmeric in the treatment of many pathogenic conditions, such as Alzheimer’s disease, Parkinson’s disease, cardiovascular diseases, and knee osteoarthritis [9,10,11,12,13].

One of the main technological operations during the spice production process is the drying of spice plants. Herbs can be dried using various conventional thermal or non-thermal technologies. Convective drying techniques in a stream of hot air are most often used in industry due to their economic advantage and speed of drying. Freeze-drying is the best-known of the non-thermal drying techniques. Direct solar or open-air drying is the most commonly applied drying technique in tropical regions due to its simplicity and economic advantage [14]. Published investigations have indicated the diverse effects of these drying techniques on product functionality, but a limited number of studies considered celery and parsley roots and turmeric rhizomes as research subjects. In previous studies, authors investigated the impact of convective drying techniques (at temperatures of 50, 60, 70, and 85 °C) and freeze-drying on the nutritional properties of celery roots [15,16]. However, the presentation of antioxidant activity in these studies differs from that in our paper. Marić et al. [17] also examined the effects of drying celery and parsley in a dryer at temperatures of 50 °C and 70 °C, as well as freeze-drying, on color, total phenol content, and DPPH test. In our research, we additionally investigated the effects of open-air drying at room temperature and convective drying at 90 °C. Llano et al. [18] studied the impact of various drying techniques on the quality of turmeric rhizomes, including traditional sun drying, fluidized-bed drying, and drying in a dryer at 50 °C. Also, none of the mentioned authors presented the results of antibacterial and antihyperglycemic analyses. The appropriate choice of drying method is very important and depends on several factors, one of the most important being the desired characteristics of the final product.

The aims of this work were to compare the effects of convective drying at different temperatures (room temperature (RT), 60 °C (60), and 90 °C (90)) and freeze-drying (FD) on the content of the phenolic compounds; antioxidant, antibacterial, and antihyperglycemic activities; and the color of the mentioned plants. Also, statistical calculation was applied to select the optimal method of drying for each sample. The obtained information could be utilized to formulate innovative functional products with improved biological potentials.

## 2. Materials and Methods

### 2.1. Chemicals and Reagents

2,4,6-Tris(2-pyridyl)-s-triazine (TPTZ), 2,2-diphenyl-1-picryhydrazyl (DPPH), 2,2-azino-bis(3-ethylbenzothiazoline-6-sulfonic acid) diammonium salt (ABTS), Folin–Ciocalteu reagent (2N), methanol (>99%), sodium hydrogencarbonate, sodium acetate, vanillin, gallic acid, quercetine hydrat, catehin, aluminium chloride, and anhydrous ferric chloride were of analytical grade or higher and purchased from Sigma-Aldrich, Merck (St. Louis, MO, USA). Ethanol (96%) was purchased from Zorka Pharma (Šabac, Serbia). Sulfuric acid, hydrochloric acid, acetic acid, di-potassium hydrogen phosphate, and potassium dihydrogen phosphate were purchased from Lach-Ner s.r.o. (Neratovice, Czech Republic). All standards for HPLC analysis and α-glucosidase were purchased from Sigma Chemical Co. (St Louis, MO, USA). All other chemicals and solvents were of the highest analytical grade.

### 2.2. Plant Material

Fresh samples of celery and parsley (25 kg each) were purchased from a local market, and fresh samples of turmeric rhizomes (25 kg) were obtained from a local health food store. All samples were purchased in Banja Luka, Bosnia and Herzegovina (44°46′ N, 17° 11′ E). The vegetables were carefully inspected, selecting only those without any defects and of similar appearance. The samples were thoroughly washed with water, dried with paper, and then cut for drying. Turmeric rhizomes and parsley roots were cut into 3 mm thick slices, and celery was cut into 1 cm × 1 cm cubes. An average of 5 kg of each representative plant material was separated for drying in open air at room temperature (approximately 25–28 °C), at 60 °C, and 90 °C and for freeze-drying. Fresh sample of root (rhizome) was frozen at −18 °C until the moment of analysis and was used as control sample. Convective drying was carried out in an oven dryer (Memmert GmBH, Buchenbach, Germany), and freeze-drying was carried out in a freeze-dryer, model Christ Alpha 2–4 LSC, from Martin Christ (Osterode am Harz, Germany). The samples were dried to a moisture content of less than 13%.

### 2.3. Extraction Procedure

Plant material (20 g), previously ground in laboratory mill (Knifetec 1095 Sample Mill, Foss A/S, Hillerød, Denmark), was poured in 200 mL of 80% ethanol and then extracted in ultrasonic cleaning bath (WUC-A03H, Witeg Labortechnik, Wertheim, Germany) for 90 min. The resulting liquid extract, protected from light, was filtered (Whatman no. 4, Sigma-Aldrich Chemie GmbH, Taufkirchen, Germany) and then evaporated on a vacuum evaporator at maximal temperature of 40 °C. The thickened extract was poured into Petri dishes and dried in a vacuum desiccator to a constant mass. Dried extracts were dissolved in 80% ethanol and used for further analyses.

### 2.4. Methods

#### 2.4.1. Total Phenolics Content (TPh)

The content of total phenolics was determined according to the modified method of Wolfe et al. [19]. Briefly, 1.5 mL of 0.2 N Folin–Ciocalteu reagent, 1.5 mL of 7.5% NaHCO_3_, and 0.2 mL of extract solution were mixed together. After vortexing and half an hour at room temperature, the absorbance was measured at 765 nm (JenWay 6305, Beacon Road, Stone, Staffordshire, UK) with a blank test. Results are expressed as mg gallic acid equivalent per gram of dry matter of plant material (mg GAE/g_dm_).

#### 2.4.2. Total Flavonoids Content (TFl)

The content of total flavonoids was determined as previously described by Zombe et al. [20]. Briefly, a 2 mL aliquot of the extract solution was mixed with 2 mL of a 2% solution of aluminum chloride in ethanol. After an hour, absorption readings at 420 nm were taken against a blank. The results are expressed as mg of quercetin equivalent per gram of dry matter of plant material (mg QE/g_dm_).

#### 2.4.3. Total Flavonols Content (Tflol)

The content of total flavonols was determined as previously described by Formagio et al. [21]. Briefly, 1 mL of an extract solution was mixed with 1 mL of a 2% solution of aluminum chloride in ethanol and 1.5 mL of 5% sodium acetate solution. After 2.5 h of incubation at room temperature in a dark place, the absorbance was measured at 440 nm against a blank. The results are expressed as mg of quercetin equivalent per gram of dry matter of plant material (mg QE/g_dm_).

#### 2.4.4. Total Flavan-3-Ols Content (Tfl3ol)

Total flavan-3-ols were determined by the vanillin–H_2_SO_4_ test [22]. Briefly, 1 mL of the extract solution was mixed with 2.5 mL of 10% (*w*/*v*) H_2_SO_4_ in methanol and 2.5 mL of a 1% solution of vanillin in methanol. After 20 min at room temperature in a dark place, the absorbance was measured at 500 nm against a blank. Results are expressed as mg of catechin equivalent per gram of dry matter of plant material (mg CE/g_dm_).

#### 2.4.5. HPLC Analysis of Individual Phenolic Compounds

Quantification of individual phenolic compounds was performed by high-resolution liquid chromatography (High-Performance Liquid Chromatography, HPLC) on Shimadzu Prominence equipment (Shimadzu, Kyoto, Japan), which contains an LC-20AT binary pump, CTO-20A thermostat, and SIL-20A automatic dispenser connected to DAD detector. The separation was performed on a Luna C-18 RP column, 5 µm and 250 × 4.6 mm (Phenomenex, Torrance, CA, USA), which was protected by a C18 precolumn, 4 × 30 mm (Phenomenex, Torrance, CA, USA). A solvent system was used as the mobile phase—A (acetonitrile) and B (1% formic acid)—at a flow rate of 1 mL/min with the following linear gradient: 0–10 min from 10 to 25% A; 10–20 min linear increase to 60% A; and from 20 to 30 min, linear increase to 70% A. The column was equilibrated to the initial condition of 10% A for 10 min, with an additional 5 min for stabilization. All samples and solvents were filtered before analysis through 0.45 µm pore size membrane filters (Millipore, Bedford, MA, USA).

#### 2.4.6. DPPH Test

For assessment of antioxidant activity, evaluation of free radical scavenging effect on the 2,2-diphenyl-1-picrylhydrazyl (DPPH) radical was used, according to the method described by Liyana-Pathirana and Shahidi [23]. A volume of 2 mL of 0.135 mM DPPH solution in methanol was mixed with 2 mL of the extract solution. After stirring, the reaction mixture was left in the dark at room temperature for 30 min. The absorbance was measured at 515 nm by UV–VIS spectrophotometer (JenWay 6305, UK). The antioxidant activity of samples is expressed as IC_50_ (mg/mL or µg/mL).

#### 2.4.7. ABTS Test

The antioxidant activity of samples was determined by ABTS radical cation (2,2′-azinobis(3-ethylbenzothiazoline-6-sulfonic acid)) decolorization assay as previously described by Re et al. [24]. ABTS stock solution was prepared freshly by mixing equal volumes of 7 mM ABTS and 2.45 mM potassium persulfate and storing in the dark at 4 °C for 16 h. The working solution was obtained by diluting the ABTS radical cation stock solution with methanol to obtain an absorbance of 0.7 ± 0.02 at 734 nm. Two milliliters of working solution was mixed with two milliliters of the extract solution. After stirring, the reaction mixture was left in the dark at room temperature for 7 min. The absorbance was measured at 734 nm by UV–VIS spectrophotometer (JenWay 6305, UK). The antioxidant activity of samples is expressed as IC_50_ value (mg/mL or µg/mL).

#### 2.4.8. FRAP Test

The FRAP method is based on the ability of antioxidants to reduce the iron (III)-2,4,6-tripyridyl-S-triazine complex [Fe(III)-TPTZ]_2_]^3+^ to the intensely blue-colored complex [Fe(II)-(TPTZ)_2_]^2+^ in an acidic medium [25]. The method consists of the preparation of the following solutions: acetate buffer pH 3.6, 20 mM FeCl_3_ × 6H_2_O, and a 10 mM solution of TPTZ reagent in 40 mM HCL. The FRAP working solution was prepared immediately before analysis by mixing acetate buffer pH = 3.6, a solution of 10 mM TPTZ (made in 40 mM HCl), and 20 mM FeCl_3_ × 6H_2_O in a volume ratio of 10:1:1, respectively. The working solution was thermostated at 37 °C. The sample solution (0.4 mL) with a known concentration was mixed with the FRAP working solution (3.6 mL) and incubated for 10 min at 37 °C, after which the absorbance at 593 nm was measured against a blank (JenWay 6305, London, UK). The results are expressed as µmol of Fe^2+^ per gram of dry matter of plant material.

#### 2.4.9. Antihyperglycemic Activity

The grounded sample (5 g) was extracted in 50 mL of potassium phosphate buffer (pH = 7.00) in ultrasonic cleaning bath (WUC-A03H, Witeg Labortechnik, München, Germany) for 30 min. Liquid extract was subsequently filtered through filter paper (Whatman no. 4, St. Louis, MO, USA) up to 50 mL with buffer. Next, 100 µL of extract was mixed with 500 µL of 2 mM 4-nitrophenyl-α-D-glucopyranoside and 500 µL of α-glucosidase solution. For control sample, 500 µL of substrate solution was mixed with 100 µL of potassium-phosphate buffer (pH = 7.00) and 500 µL of enzyme. After 10 min of incubation at 37 °C, absorbance was read at 405 nm using UV–VIS spectrophotometer (JenWay 6305, UK) against blank [26]. The percentage of enzyme inhibition was calculated using the following equation:

(1) $$Inhibition\left(\%\right)=\frac{A_{control}-A_{sample}}{A_{control}}\times 100$$

#### 2.4.10. Antibacterial Activity

The antibacterial activities of turmeric, celery, and parsley extracts were tested by the disk diffusion method. Celery and parsley extracts were dissolved in water, and turmeric extracts in 40% ethanol to concentration of 100 mg/mL. The test microorganisms were the Gram-negative bacterium *Escherichia coli* ATCC 25922 and the Gram-positive bacterium *Staphylococcus aureus* ATCC 25923. Bacterial strains were grown on Müeller–Hinton slant (Himedia, Mumbai, India) for 24 h at 37 °C. Cells were then suspended in a sterile 0.9% NaCl solution. The suspension for inoculation was adjusted to a concentration of 1 × 10^6^ cfu/mL, which was estimated using Densichek (Biomerieux, France). The suspensions (1 mL) for inoculation were homogenized with melted (45 °C) Müeller–Hinton agar (9 mL) and poured into Petri dishes. Sterile 6 mm discs (Himedia, Mumbai, India) were placed on the inoculated agar plates and impregnated with 15 µL of extract solution. The antibiotic discs (30 µg cefotaxime/10 µg clavulanic acid per disc, Bioanalyse^®^, Ankara, Turkey) were used as positive control and distilled water and 40% ethanol as negative control (15 µL). The test plates were refrigerated at 8 °C for 1 h to allow the extracts to diffuse into the medium, and then were incubated for 24 h at 37 °C. After the incubation, the diameters of the inhibition zones were measured and recorded in millimeters (mm). The evaluation of antibacterial activity was carried out in three repetitions.

#### 2.4.11. Color Measurements

The chromaticity indices (L*, a*, and b* values) of fresh and dried slices of roots and rhizomes were measured in triplicate using a Minolta chromameter, HunterLab Europe GmbH, Murnau, Germany (model CM 2600d, Osaka, Japan). CIE L*a*b* color space difference represents color difference perceived by humans. Results are expressed as mean of minimum 8 replicates.

#### 2.4.12. Artificial Neural Network (ANN) Model

For the ANN modeling phase, which included 15 samples, the data were divided into training (60%), cross-validation (20%), and testing (20%) sets. To enhance accuracy, min–max normalization was applied to standardize both input and output data. The proposed multilayer perceptron ANN model (MLP) featured a three-layer architecture with feedforward design and utilized backpropagation training, as discussed in previous studies [27,28]. The hidden layer consisted of 5 to 10 neurons, and various activation functions (tangent, sigmoidal, exponential, and identity) were tested. The BFGS algorithm was employed to construct the ANN model, iteratively adjusting weights and biases across 100,000 different configurations. The objective was to minimize square error until both the learning and cross-validation curves approached zero.

The model’s accuracy underwent evaluation through a range of standard computational tests, including the coefficient of determination (r^2^), reduced chi-square (χ^2^), mean bias error (MBE), root mean square error (RMSE), mean percentage error (MPE), sum of squared errors (SSE), and average absolute relative deviation (AARD) [29].

#### 2.4.13. Standardized Scores

The ranking for 15 samples involved comparing raw data with extreme values, following the methodology outlined by Brlek et al. [30]. Criteria for ranking were based on parameters such as total phenolic, total flavonoids, total flavonols, total flavan-3-ols contents, FRAP antioxidant test, as well as lightness L* (for all samples), a* and b* values (for turmeric samples) with a preference for higher values, and IC_50_^DPPH^, IC_50_^ABTS^ (for all samples), a* and b* values (for celery and parsley samples) with a preference for lower values.

#### 2.4.14. Statistical Analysis

The data were statistically analyzed using the STATISTICA 10.0 software package (StatSoft Inc., Tulsa, OK, USA). All analyses were performed in triplicate. The results were presented as mean values with standard deviations (SD). Variance equality was assessed using Levene’s test, and normal distribution was checked using the Shapiro–Wilk test. Results were subjected to a one-factor analysis of variance (ANOVA) followed by Tukey’s HSD post hoc test.

## 3. Results and Discussion

### 3.1. Phenolic Compounds

The contents of phenolic compounds are shown in Table 1. The contents of total phenolics in the samples CF, PF, and TF were 1.68 mg GAE/g_dm_, 1.50 mg GAE/g_dm_, and 32.18 mg GAE/g_dm_, respectively. The content of total phenolics in fresh celery root was slightly lower compared to the research by Salamatullah et al. [31] and Prieciņa and Kārkliņa [32], who reported the values of 2.2 mg GAE/g and 3.3 mg GAE/g, respectively. Our values were higher than those reported by the authors of Yao et al. [33], which were in the range of 3.48–5.02 mg GAE/100 g_dm_ for different celery varieties. These differences in the content of phenolic substances may be the result of different methods of extraction, as well as genotypes, agroclimatic conditions, picking time, storage methods, and so on [34]. The content of total phenolics was the highest in the CF sample and was statistically significantly different from the other treated samples (*p* < 0.05), while among the dried samples, the highest content of these compounds was found in the C60 sample, which was not statistically significantly different from the C90 sample (*p* > 0.05). Our results were in accordance with the research of Liang et al. [35], which confirmed that the convective drying method achieves a higher content of total phenolics compared to freeze-drying. This occurrence could be explained by the synthesis or release of phenolic compounds during drying or by the action of enzymes released from the damaged plant tissue, which catalyze the reactions of oxidation, hydrolysis, or glycolysis. For these reactions to take place, an elevated temperature is required. This phenomenon was also observed by Miao et al. [36]. Additionally, Roshanak et al. [37] found that fresh and freeze-dried green tea samples had lower levels of phenolic compounds and antioxidant activity (measured by DPPH) compared to samples dried at 60 °C. Also, in a study conducted by Skendi et al. [38], it was found that most of the freeze-dried samples had the lowest levels of total phenolic compounds compared to those dried using other techniques. It was also noticed that with an increase in drying temperature, there was an increase in the content of total phenolics in the C60 sample compared to the CRT sample, and this conclusion was in accordance with the research of Kręcisz et al. [16]. This effect could be explained by the possible binding of polyphenols to other compounds or a change in their chemical structure as a result of degradation [15]. The content of total flavonoids, flavonols, and flavan-3-ols was also the highest in the fresh sample of celery. A decrease in the content of flavonoids, flavonols, and flavan-3-ols after convective drying of celery roots was also reported by Prieciņa and Kārkliņa [32], while the lowest content of these compounds was found in the CFD sample. Among the dried samples, the content of total flavonols was the highest in the C60 sample, followed by the CRT sample (*p* < 0.05), while the lowest content was in the C90 sample, probably due to their lack of resistance to higher temperatures. The content of total flavonoids and flavan-3-ols decreased with increasing drying temperatures, while the content of flavonols decreased above 60 °C. The higher content of total phenols in the sample C60 can be explained by the fact that the enzyme polyphenol oxidase is inactivated at that temperature [39], while further increasing the temperature leads to thermal degradation of the structure of these compounds, which causes a decrease in their concentration. The content of total phenolics was the highest in the PRT sample and was statistically significantly different from the other samples (*p* < 0.05). The content of these compounds in the fresh sample was lower compared to the results reported by Dobričević et al. [40] (2.24–3.35 mg GAE/g_dm_ depending on the tested variety). Similar to celery, the freeze-dried sample of parsley root showed the lowest content of total phenols, which was not statistically significantly different from the P90 sample (*p* > 0.05). A lower content of these compounds was also found in the P90 sample compared to the PF, PRT, and P60 samples (*p* < 0.05). The content of total flavonoids and flavan-3-ols was the highest in the fresh sample of parsley (*p* < 0.05). Among the treated samples, the flavonoid content was the highest in the P60 sample and the lowest in the PFD and PRT samples (*p* > 0.05). With the convective drying technique, the flavan-3-ol content decreased with increasing temperature and was the lowest in the P90 sample and the highest in the PRT sample.

Unlike the previous two samples, the content of total phenolics was the highest in the freeze-dried turmeric sample (*p* < 0.05) and the lowest in the T90 sample, which was not statistically significantly different from the control sample (*p* > 0.05). The content of these compounds was higher at lower drying temperatures, probably due to the lower resistance of these compounds to elevated temperatures. Similar observations were also observed by the authors of Lima et al. [41], where there was a reduction of these compounds in the sample dried at 75 °C compared to the sample dried at 65 °C. The content of these compounds in the control sample was slightly lower compared to the values reported by Komonsing et al. [42], which were in the range of 34.9–53.4 mg GAE/g. The authors used methanol for extraction. Our results for the TRT sample were higher compared to the authors’ research [43] (26.37 mg GAE/g or 28.88 mg GAE/g_dm_). The content of total flavonoids, flavonols, and flavan-3-ols was also the highest in the freeze-dried turmeric sample, and, in general, their content was higher at lower drying temperatures, except for total flavonoids and flavan-3-ols, whose concentrations were higher in the T90 sample compared to sample T60. This difference in total flavonoids was not statistically significant (*p* > 0.05). The increase in the content of phenolic compounds in the TRT sample compared to the CRT and PRT samples may be due to the inhibitory effect of curcumin on polyphenol oxidase, whereby the oxidation of phenolic compounds is reduced [44]. Also, the higher content of these compounds in the freeze-dried turmeric sample compared to other turmeric samples could be explained by the fact that during this process, small pores are formed inside the sample due to the formation of small ice crystals and their sublimation, which later enables a higher extraction efficiency, which ultimately leads to a higher content of these compounds [45,46]. The authors of Chumroenphat et al. [47] also reported higher levels of total phenolic compounds in freeze-dried turmeric rhizome compared to samples that were dried using hot air or sun-drying methods. However, other authors state that TPC and antioxidant capacity can either increase or decrease after drying, depending not only on the variety but also on the production system used (conventional or organic) [48].

### 3.2. Antioxidant Activities

Antioxidant activities of samples are presented in Table 2. It can be noted that all treated samples showed higher antioxidant activity with respect to the DPPH test compared to the control sample. The CRT sample showed the highest antioxidant activity among the treated celery samples, while the freeze-dried sample showed the worst antioxidant activity. Considering the DPPH and FRAP tests, all treated celery samples showed better antioxidant activities compared to the control sample, while in the ABTS test, the control sample showed the highest antioxidant potential. Freeze-dried celery samples had a similar antioxidant capacity as the control sample and did not differ statistically significantly according to the DPPH and FRAP tests (*p* > 0.05). These differences could be explained by the different mechanisms of the reaction of free radicals and Fe^2+^ ions with antioxidants. Namely, the ABTS test is based on the proton transfer mechanism (HAT), the FRAP test on single electron transfer (SET), and the DPPH on the combination of the previous two HAT/SET mechanisms [49].

The higher antioxidant power of dried samples could be explained by the fact that during drying, phenolic compounds are oxidized and have a higher antioxidant power than their non-oxidized forms or new compounds are formed that show antioxidant activity, e.g., during the Maillard reaction [50,51]. The decrease in the content of phenolic compounds with the simultaneous increase in the antioxidant activity may be the result of several factors, such as the increased antioxidant power of phenols in the transition state of oxidation, the increase in the content of reducing sugars, and the formation of Maillard reaction products (MRP), which are known to have high antioxidant activity [52].

Our results for freeze-dried turmeric for the DPPH test were much lower (higher antioxidant activity) compared to the results reported by Tiveron et al. [53] (IC_50_^DPPH^ = 21.14 mg/mL), and for the FRAP test, the results were significantly higher (169.1 µmol Fe^2+^/g_dm_). Furthermore, our turmeric sample showed a better antioxidant capacity compared to the methanol extract of lyophilized turmeric (IC_50_^DPPH^ = 600.7 µg/mL) [54]. Comparing the IC_50_ values for the DPPH and ABTS tests for TRT, T60, and T90 samples, it is evident that lower drying temperatures contribute to greater preservation of the antioxidant capacity of the samples. A similar conclusion was confirmed by the authors of Lima et al. [41]. Regarding the ABTS and FRAP tests, an increase in the drying temperature was accompanied by a decrease in the antioxidant activity, while in regard to the DPPH test, an increase in the antioxidant activity was observed in the T90 sample compared to the T60 sample, but without a statistically significant difference (*p* > 0.05).

The PRT sample, with regard to the ABTS and FRAP tests, showed the strongest antioxidant activity and was statistically significantly different from other samples (*p* < 0.05). Considering the results of the DPPH and FRAP tests, it can be concluded that increasing the drying temperature leads to a decrease in the antioxidant activity of these samples (*p* > 0.05), while according to the ABTS test, sample P90 showed slightly more pronounced antioxidant activity compared to P60. The control sample and PFD, according to the FRAP test, were not statistically significantly different.

### 3.3. Color Analysis

As shown in Table 3, in all freeze-dried samples, a statistically significant difference was observed between the parameters of brightness (L*) and chromaticity (a* and b*) compared to the fresh sample. Specifically, a significant increase in the brightness of the freeze-dried samples was recorded, which is in accordance with the results of Kręcisz et al. [16] and Marić et al. [17] for celery and parsley samples, respectively. The decrease in the value of a* and the increase in the value of b* in CFD and PFD samples were also in accordance with the research of Marić et al. [17] for freeze-dried parsley, while the authors of Kręcisz et al. [16] reported a slight increase in the value of the parameter a*. Furthermore, it is evident that in the CRT and PRT samples, there was the greatest increase in the values of parameters a* and b*, while in the TRT sample, there was a decrease in the values of these parameters. Such differences between the color and brightness parameters of freeze-dried samples and open air-dried samples can be explained by the fact that freeze-drying leads to the sublimation of ice crystals and less exposure to oxygen, and due to low temperatures, the deactivation of polyphenol oxidase occurs, which prevents enzymatic browning reactions [55].

### 3.4. Correlation Analysis

The results of the correlation analysis of phenolic compounds and antioxidant activity are shown in Table 4. There was a very strong statistically significant positive correlation between total phenolic and total flavonoid contents with a correlation coefficient of 0.994 (*p* = 0.000). Similarly, there was a very strong statistically significant positive correlation between total phenolic and total flavonol contents with a correlation coefficient of 0.984 (*p* = 0.000). Total phenolic content also showed a very strong statistically significant positive correlation with the total flavan-3-ol content with a correlation coefficient of 0.982 (*p* = 0.000). On the other hand, there was a very strong, statistically significant, negative correlation between IC_50_^ABTS^ and FRAP with a correlation coefficient of −0.904 (*p* = 0.000). Additionally, the FRAP values exhibited an extremely strong, positive, statistically significant correlation with the total phenolic content with a correlation coefficient of 0.999 (*p* = 0.000). Total phenolic content had a strong, negative, statistically significant correlation with IC_50_^ABTS^ (r = −0.910, *p* = 0.000) and a moderately strong, negative, statistically significant correlation with IC_50_^DPPH^ (r = −0.759, *p* = 0.001). Total flavonoids also showed a strong, negative, statistically significant correlation with IC_50_^ABTS^ (r = −0.889, *p* = 0.000) and a moderately strong, statistically significant, negative correlation with IC_50_^DPP^H (r = −0.744, *p* = 0.001). Total flavan-3-ols showed a strong, positive, statistically significant correlation with FRAP (r = 0.982, *p* = 0.000).

### 3.5. Cluster Analysis

**Figure 1:**
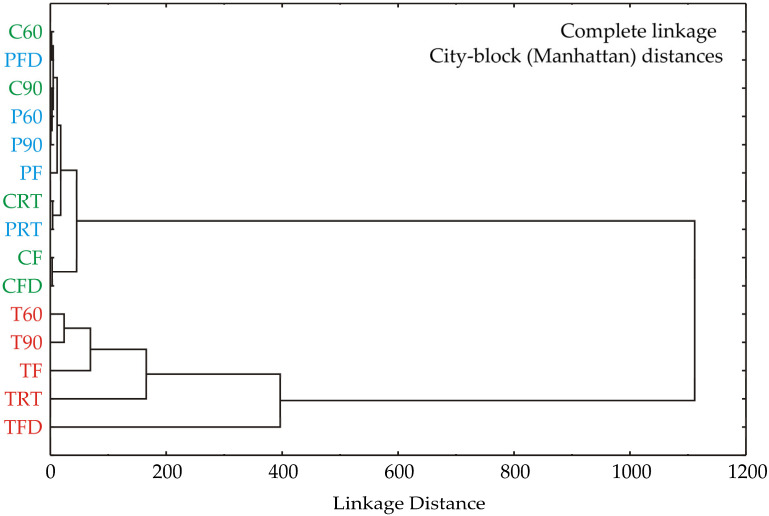
Cluster analysis of celery (green color), parsley (blue), and turmeric (red) samples according to differences in total phenolic, total flavonoid, total flavonol, and total flavan-3-ol contents and IC_50_^ABTS^, IC_50_^DPPH^, and FRAP antioxidative tests.

The cluster analysis conducted on the samples revealed two main clusters (Figure 1). In the first cluster were the following celery and parsley samples: C60, C90, CF, CRT, CFD, P60, P90, PF, PRT, and PFD. The second cluster comprised turmeric samples T60, T90, TF, TRT, and TFD. The linkage distance between these clusters was close to 1100.

The hierarchical cluster analysis using complete linkage and City-block (Manhattan) distances reveals distinct groupings among the variables. The dendrogram shows two main clusters. The first major cluster, containing C60, PFD, C90, P60, P90, PF, CRT, PRT, CF, and CFD, merges at a linkage distance of approximately 400. Within this cluster, there are several subgroups: C60 and PFD exhibit the highest similarity, merging at a very low distance, followed by C90, P60, and P90. The remaining variables in this group (CRT, PRT, CF, and CFD) show moderate similarity with slightly larger distances.

The second major cluster includes the variables T60, T90, TF, TRT, and TFD, which merge at a greater distance, close to 1000, indicating less similarity between these variables and the others. Within this cluster, T60, T90, and TF are closely related, merging at around 200, while TRT and TFD are more distinct but still part of the same general grouping.

The large gap between the two main clusters, reflected in the significant linkage distance (~1000), suggests that the underlying data for these two groups of variables are considerably different. This separation may indicate two distinct patterns or characteristics in the dataset. Within the groups, the tight clustering of certain variables, such as C60 with PFD and T60 with T90, indicates high similarity and potentially shared underlying factors.

### 3.6. Principal Component Analysis (PCA)

**Figure 2:**
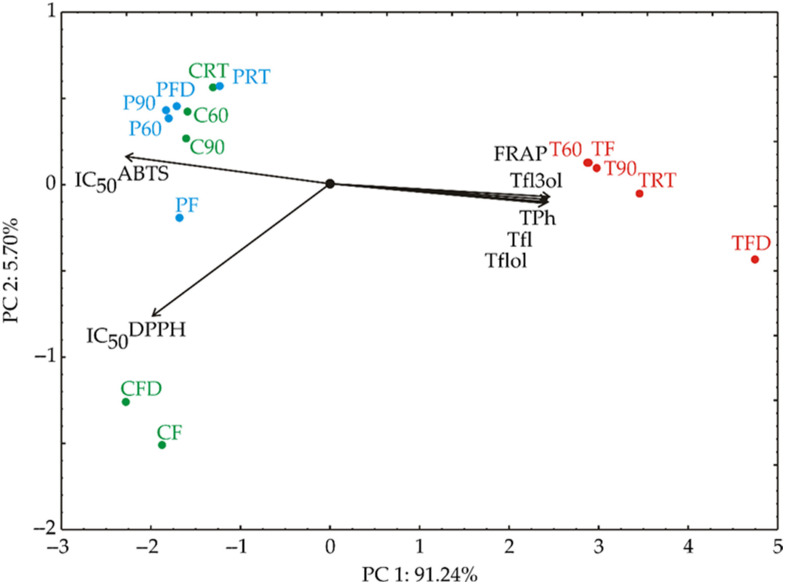
The PCA biplot diagram, of celery (green color), parsley (blue), and turmeric (red) samples, depicting the relationships among total phenolic, total flavonoid, total flavonol, and total flavan-3-ol contents, as well as the IC_50_^ABTS^, IC_50_^DPPH^, and FRAP antioxidative tests.

Principal Component Analysis (PCA) was utilized to explore the interrelationships among various samples, as depicted in Figure 2. In this analysis, the proximity of points on the PCA graph signifies similarity in patterns [56]. The direction of vectors in the factor space reveals variable trends, with the length of vectors indicating the strength of correlation [57]. The first two principal components (PCs) elucidated a substantial portion, explaining 96.94% of the total variance in the dataset. Specifically, the first PC accounted for 91.24%, while the second PC explained 5.70% of the overall variance within the data collected.

When examining the projection of variables onto the factor plane, notable positive contributions to the first principal component (PC1) were observed: TPh (15.49% based on correlation), Tfl (15.38%), Tflol (15.07%), Tfl3ol (15.15%), and FRAP (15.52%). The most negative influence on the PC1 component was noticed for IC_50_^ABTS^ (13.31%) and IC_50_^DPPH^ (10.08%). Conversely, IC_50_^DPPH^ (89.14%) made significant negative contributions to the second principal component (PC2), as depicted in Figure 2.

The biplot (Figure 2) displays variable vectors (IC_50_^DPPH^, IC_50_^ABTS^, FRAP, TPh, Tfl, Tfl3ol, and Tflol) and sample groupings. Variables with longer vectors (such as IC_50_^DPPH^, IC_50_^ABTS^, and FRAP) contribute more to the variation along the respective principal component axis. The direction of the vectors shows the relationship between the variables and their influence on the sample distribution.

The first cluster (T60, T90, TF, TRT, and TFD) is positively associated with variables such as FRAP, TPh, Tfl, Tflol, and Tfl3ol, suggesting that these samples have high values for total phenolics, total flavonoids, and related antioxidant activities. The second cluster (C60, C90, P60, P90, PFD, CRT, PRT, and PF) is positioned along the negative axis of PC1, indicating a strong association with the IC_50_ values for ABTS and DPPH, suggesting these samples have relatively higher free radical scavenging activities. The third cluster (CFD and CF) is located near the lower-left quadrant and is strongly influenced by the IC_50_^DPPH^ vector, indicating these samples have a distinctive antioxidant profile dominated by the DPPH scavenging activity.

The separation of the clusters highlights distinct differences in the chemical or antioxidant profiles of the samples, with the samples on the right (first cluster) being rich in phenolics and flavonoids, while the samples on the left (second and third clusters) showing strong antioxidant capacities as measured by the ABTS and DPPH assays. This clustering indicates that the variables selected for analysis effectively differentiate the samples based on their bioactive properties.

### 3.7. Standardized Scores

**Figure 3:**
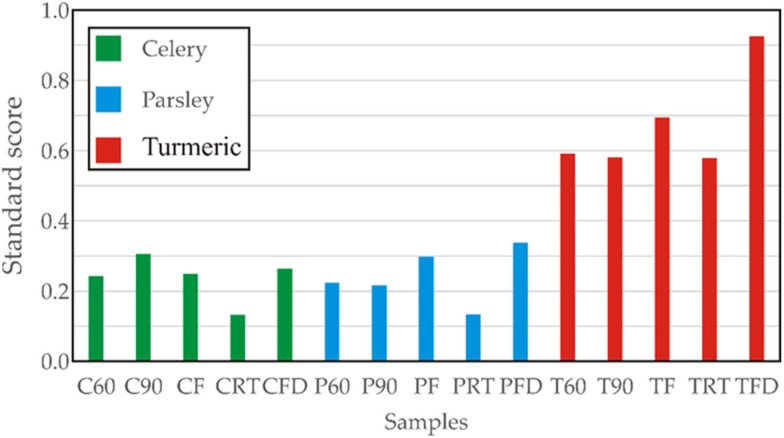
Standard scores for control and dried celery, parsley, and turmeric samples.

The standardized score (SS) is calculated by summing up the normalized scores of each variable (total phenolic, total flavonoid, total flavonol, total flavan-3-ol, IC_50_^ABTS^, IC_50_^DPPH^, FRAP, and chromaticity indices). Maximizing the SS function allows for the determination of the optimal drying method. As shown in Figure 3, samples C90, PFD, and TFD had the highest SS values.

The standardized score analysis of bioactive compounds across celery, parsley, and turmeric samples reveals significant differences in concentration levels of total phenolic, total flavonoid, total flavonol, and total flavan-3-ol contents, as well as the IC_50_^ABTS^, IC_50_^DPPH^, and FRAP antioxidative tests and the color parameters L*, a*, and b*. Turmeric samples exhibit the highest standard scores, with TFD showing the most pronounced value (standard score ≈ 0.95), indicating its dominance in bioactive compound concentration and antioxidant profile. The other turmeric samples, such as TRT and TF, also display high scores (≈0.6–0.7). In contrast, parsley samples show lower scores, with PF achieving higher value (≈0.2), while PRT, P60 and P90 exhibiting lower scores (≈0.2–0.3). Among celery samples, CRT has the lowest score, followed by CF, CFD and C60 (≈0.2). These findings highlight that turmeric samples, particularly TFD, contain significantly higher concentrations of bioactive compounds compared to both celery and parsley samples.

### 3.8. Artificial Neural Network (ANN) Model

**Figure 4:**
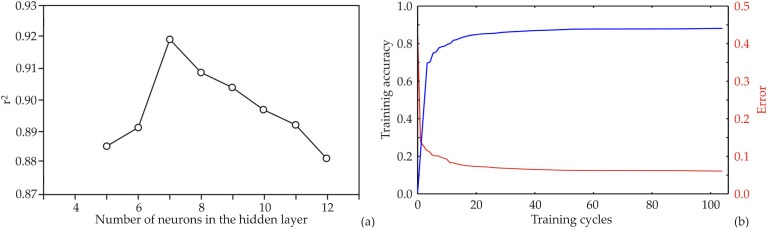
ANN calculation: (**a**) The dependence of the r^2^ value of the number of neurons in the hidden layer in the ANN model, (**b**) Training results per epoch (the training accuracy was presented by blue line, while the error was presented by red line).

The structure and outcomes of the artificial neural network (ANN) model are significantly influenced by the initial assumptions regarding matrix parameters, such as biases and weights. These initial assumptions are crucial for accurately fitting the model to the experimental data. Additionally, the model’s performance is affected by the number of neurons in the hidden layer. To mitigate the impact of random correlations from initial assumptions and weight initialization, the model was run 100,000 times with randomized topologies. The optimal model, yielding the highest r^2^ value, was achieved with seven hidden neurons (Figure 4a). Each ANN model underwent training for 100 epochs. Training accuracy increased with each cycle, stabilizing around the 70th to 80th epoch. Training beyond 80 epochs could lead to significant overfitting, whereas 80 epochs were sufficient to achieve high model accuracy without overfitting (Figure 4b).

The optimized neural network models exhibited robust generalization capabilities for the experimental data, effectively predicting outputs based on the input parameters. Utilizing a configuration of seven neurons (network MLP 8-7-7), the ANN model achieved high r^2^ values, specifically 0.917 for training, 1.000 for testing, and 0.998 for validation. The training error was 214.001, the test error was 412.417, and the validation error reached 414.368. The used training algorithm was BFGS 122, while the error function was sum of squares. The hidden activation function was tanh, while the output activation was logistic.

**Figure 5:**
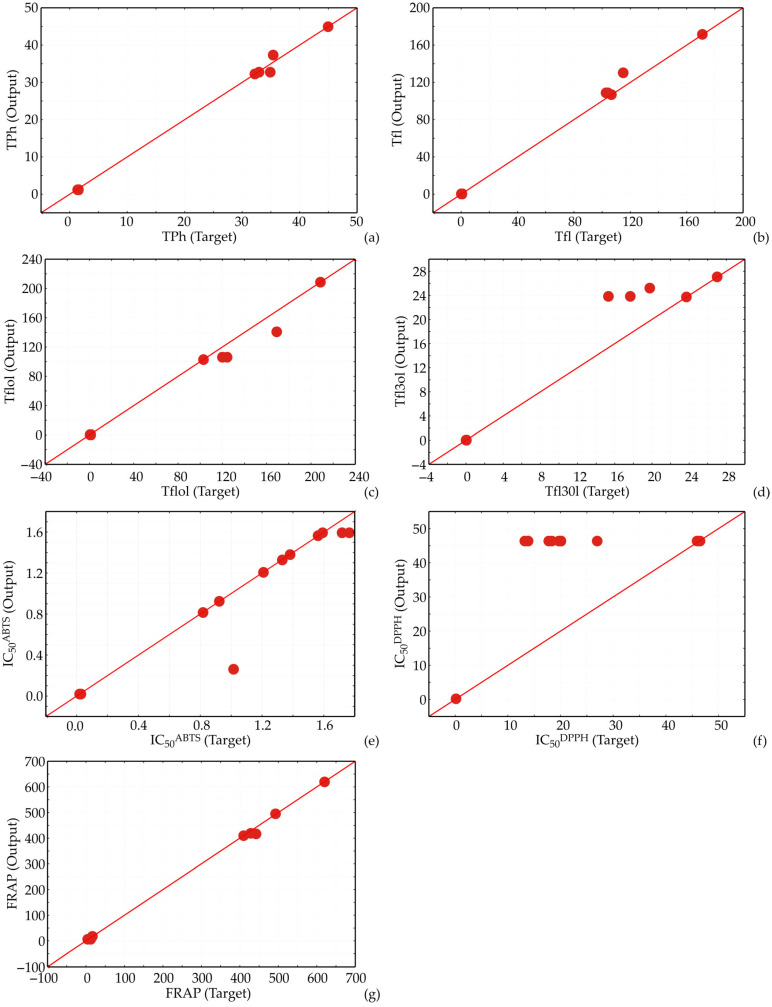
Experimental and predicted values obtained for (**a**) total phenolic, (**b**) total flavonoid, (**c**) total flavonol, (**d**) total flavan-3-ol, (**e**) IC_50_^ABTS^, (**f**) IC_50_^DPPH^, and (**g**) FRAP.

The ANN model effectively captured the underlying relationships between the input variables and their respective outputs, reinforcing their robustness in predicting both adhesion inhibition and biofilm formation. This high level of agreement suggests that the ANN approach is well suited for complex biological processes, where nonlinear interactions are prevalent. Furthermore, the consistent performance across training, testing, and validation phases indicates the models’ generalization capability, making them reliable for broader applications in predicting inhibitory effects under various conditions (Figure 5).

The model’s feature fit was assessed, and the results are presented in Table 5, showing that the ANN models exhibited a minor lack of fit tests, indicating satisfactory prediction of the analyzed parameters. The r^2^ values for total phenolic, total flavonoid, total flavonol, total flavan-3-ol, IC_50_^ABTS^, IC_50_^DPPH^, and FRAP predictions were 0.998, 0.997, 0.991, 0.958, 0.920, 0.591, and 0.999, respectively, suggesting that the models accurately evaluated variations and fit the data well, excluding the prediction of the IC_50_^DPPH^ value.

For the content of total phenolic, the model demonstrated excellent accuracy with an r^2^ value of 0.998, indicating a very high degree of fit to the experimental data. The errors were low, with an RMSE of 0.810 and an MBE of 0.227. The mean percentage error was 13.937%, which is relatively modest. The distribution of errors was slightly left-skewed with a high kurtosis, suggesting a distribution with a pronounced peak. In the case of the content of total flavonoids, the model also showed high accuracy, with an r^2^ value of 0.997. However, the percentage errors were larger, with an MPE of 43.590% and an AARD of 81.732. This model’s error distribution was highly left-skewed with a significant peak, indicating more considerable deviations from the mean in one direction. For total flavonols, the model maintained a good fit with an r^2^ of 0.991. The residuals were higher, reflected by an RMSE of 9.310 and a χ^2^ of 92.861. The error distribution was right-skewed with moderate kurtosis, indicating that most errors were on one side of the mean, with a distribution that is neither too peaked nor too flat.

The model for content of total flavan-3-ols also performed well with an r^2^ of 0.958, indicating good accuracy. The errors were higher, with an MPE of 47.921% and an AARD of 89.852. The distribution of errors was left-skewed with moderate kurtosis, suggesting that errors were more frequently on one side of the mean. For IC_50_^ABTS^, the model showed reasonable accuracy with an r^2^ of 0.920. The errors were low, with an RMSE of 0.205 and a χ^2^ of 0.045. The error distribution was right-skewed with a sharp peak, indicating that most errors were positive and closely clustered around the mean. Conversely, the model for IC_50_^DPPH^ showed poor predictive performance, with an r^2^ of only 0.591, indicating a lower degree of fit to the experimental data. The errors were high, with an RMSE of 20.434 and an MPE of 94.546%. The error distribution was symmetrical but flatter than a normal distribution, suggesting significant variability in the predictions. Finally, the model for FRAP exhibited outstanding predictive accuracy with an r^2^ of 0.999. The errors were minimal, with an RMSE of 7.313 and an MPE of 9.474%. The error distribution was right-skewed with a significant peak, indicating that the errors were mostly positive and tightly clustered around the mean. In summary, while the models for total phenolic, total flavonoid, total flavonol, total flavan-3-ol, and FRAP showed high accuracy and good fit, the IC_50_^DPPH^ model significantly underperformed. The analysis of error distributions further highlighted the variability and reliability of these models, indicating that most models could reliably predict the experimental parameters, with the notable exception of IC_50_^DPPH^.

### 3.9. HPLC Analysis of Phenolic Compounds and the Antihyperglycemic and Antimicrobial Activity of Selected Samples

The analysis of the content of individual phenolic compounds was performed using the HPLC method in the selected samples, which showed the highest SS values. Results are shown in Table 6. Gallic and *p*-coumaric acids were the most abundant of the tested phenolic acids in the C90 sample, while in the PFD sample, ferulic acid was dominant, which was in accordance with the research of Arsenov et al. [58], and then protocatechuic acid. The TFD sample contained a larger number of different phenolic compounds compared to the other two samples, but in a smaller amount. In this sample, catechin derivatives, i.e., catechin and epicatechin, and phenolic acids, i.e., protocatechuic acid, were dominant. Phenolic acids are characterized by rapid absorption in the human body, and many beneficial effects that these substances contribute to human health are known [59]. In the C90 and PFD samples, a significant peak was observed on the chromatogram at a retention time that corresponds to apiin; similarly, in the TFD sample, peaks at retention times that correspond to curcuminoids, characteristic compounds for turmeric, were observed, which could not be quantified due to the lack of standards.

As it could be observed from Table 6, selected celery, parsley, and turmeric samples contained a variety of different phenolic compounds known to be beneficial for human health. Those compounds remained in the investigated samples after the application of drying technique and can be used as spices, during cooking, or in the food industry.

Inhibition of α-glucosidase transforms the management of post-prandial glucose levels by delaying carbohydrate absorption, thereby supporting better overall glycemic control and reducing the risk of hyperglycemia in individuals with diabetes [60]. Antihyperglycemic activities of selected samples are shown in Table 7. The strongest potential to inhibit α-glucosidase enzyme was shown by the turmeric sample, and our result was in line with previously published research [61]. The turmeric sample had the highest concentration of total phenolics compared with the two other tested samples. Previous studies have confirmed a high correlation between the total phenolic content and α-glucosidase inhibitory activity [62,63]. The PFD sample at the same concentration showed an approximately 5-fold weaker potential to inhibit this enzyme. C90, at a concentration of 40 mg/mL, did not show any antihyperglycemic activity, but at a concentration of 100 mg/mL, it inhibited 84.44% of the enzyme.

Many plant secondary metabolites, such as phenolic acids and flavonoids, are known to exhibit antimicrobial activity. They achieve this activity through different mechanisms by affecting protein synthesis, metabolic processes in the cell wall of bacteria, or inhibiting the synthesis of ATP and DNA [64,65]. The antibacterial properties of the extracts of selected celery, parsley, and turmeric samples are shown in Table 8.

Among the tested samples, only the TFD sample showed antibacterial activity against *S. aureus*. This result is consistent with previous research. The authors of Singh et al. [66] reported lower antibacterial activity compared to the value of the ethanolic extract of turmeric rhizome at a concentration of 100 mg/mL in our study. Also, the authors of Wada et al. [67] reported a higher value of the zone of inhibition (13 mm) for the ethanolic extract of turmeric rhizome at a concentration of 100 mg/mL. The TFD sample did not show antibacterial activity against *E.coli*, which was in line with the research of Wada et al. [67]. Gram-negative bacteria are less susceptible to the influence of plant extracts due to the presence of an outer membrane, which contains lipopolysaccharides and represents a protective barrier preventing the diffusion of toxic and hydrophobic substances [68,69]. Samples C90 and PFD did not show any antibacterial activity against the tested microorganisms, probably due to a significantly lower content of phenolic compounds in these samples compared to the TFD sample.

## 4. Conclusions

In this study, different drying techniques (open air at room temperature, convective drying at 60 °C and 90 °C, and freeze-drying) were applied on celery, parsley, and turmeric plants. The best drying methods, in terms of preserving phenolic compounds, were convective drying of celery roots at 60 °C, open-air drying at room temperature of parsley roots, and freeze-drying of turmeric rhizomes. Celery and parsley samples dried in open air at room temperature showed the best antioxidant activities, while freeze-dried turmeric rhizomes showed the best antioxidant potential. On the other hand, a very important characteristic in food technology is the preservation of the sample color in order to increase the sensory acceptability of the final product. In this sense, the celery sample dried at 90 °C as well as the freeze-dried turmeric rhizomes and parsley roots gave the best results for color parameters, which were most similar to their control samples. CRT and PRT samples showed the best antioxidant activities, while the TFD sample showed the best antioxidant potential. Considering all the mentioned characteristics and applied statistical calculation, the optimal drying technique was convective drying at 90 °C for celery roots and freeze-drying for parsley roots and turmeric rhizomes. Also, the TFD extract showed good antimicrobial activity against *Staphylococcus aureus* but not against *Escherichia coli*. C90 and PFD extracts did not show antimicrobial activity against the tested microorganisms. Since a small number of scientific studies have been performed on the different drying techniques of celery and parsley roots and turmeric rhizomes and their influence on biological activities, this work represents a certain contribution in this field and suggests the usage of dried spices as good sources of bioactive compounds as part of the everyday diet or in the food industry.

## Figures and Tables

**Table 1:** Contents of total phenolics, total flavonoids, total flavonols, and total flavan-3-ols in control and dried samples.

Sample	Total Phenolics (mg GAE/g_dm_)	Total Flavonoids (mg QE/g_dm_)	Total Flavonols (mg QE/g_dm_)	Total Flavan-3-Ols (mg CE/g_dm_)
CF	1.68 ± 0.05 ^d^	0.15 ± 0.007 ^d^	1.40 ± 0.06 ^e^	0.24 ± 0.00 ^e^
CRT	1.31 ± 0.05 ^b^	0.14 ± 0.01 ^c^	0.73 ± 0.04 ^c^	0.11 ± 0.00 ^d^
C60	1.44 ± 0.04 ^c^	0.10 ± 0.00 ^b^	0.82 ± 0.02 ^d^	0.08 ± 0.00 ^c^
C90	1.40 ± 0.04 ^bc^	0.10 ± 0.00 ^b^	0.49 ± 0.01 ^a^	0.06 ± 0.00 ^b^
CFD	1.13 ± 0.03 ^a^	0.06 ± 0.00 ^a^	0.60 ± 0.01 ^b^	0.05 ± 0.00 ^a^
PF	1.50 ± 0.04 ^b^	0.75 ± 0.01 ^d^	1.46 ± 0.02 ^b^	0.08 ± 0.00 ^e^
PRT	1.58 ± 0.03 ^c^	0.53 ± 0.01 ^a^	1.51 ± 0.03 ^b^	0.05 ± 0.00 ^d^
P60	1.48 ± 0.01 ^b^	0.60 ± 0.00 ^c^	1.56 ± 0.06 ^b^	0.03 ± 0.00 ^c^
P90	1.38 ± 0.01 ^a^	0.56 ± 0.00 ^b^	1.77 ± 0.05 ^c^	0.01 ± 0.00 ^a^
PFD	1.32 ± 0.02 ^a^	0.52 ± 0.01 ^a^	0.80 ± 0.01 ^a^	0.02 ± 0.00 ^b^
TF	32.18 ± 1.05 ^a^	106.55 ± 0.76 ^a^	103.00 ± 6.55 ^a^	23.73 ± 0.42 ^d^
TRT	35.43 ± 0.36 ^b^	114.86 ± 3.77 ^a^	169.22 ± 5.69 ^c^	19.74 ± 0.65 ^c^
T60	34.91 ± 0.51 ^b^	102.58 ± 4.25 ^a^	124.21 ± 6.55 ^b^	15.29 ± 0.79 ^a^
T90	32.94 ± 0.05 ^a^	104.14 ± 6.75 ^a^	119.86 ± 0.35 ^b^	17.67 ± 0.36 ^b^
TFD	44.92 ± 1.27 ^c^	171.26 ± 6.43 ^b^	208.72 ± 1.02 ^d^	27.04 ± 1.06 ^e^

Note: The results are presented as mean value ± standard deviation (*n* = 3). ^a–e^ Mean values with different letters in superscript in the same column among each plant sample are statistically different according to the Tukey’s HSD test (*p* < 0.05). CF—celery fresh, CRT—celery open-air dried at room temperature, C60—celery dried at 60 °C, C90—celery dried at 90 °C, CFD—celery freeze-dried, PF—parsley fresh, PRT—parsley open-air dried at room temperature, P60—parsley dried at 60 °C, P90—parsley dried at 90 °C, PFD—parsley freeze-dried, TF—turmeric fresh, TRT—turmeric open-air dried at room temperature, T60—turmeric dried at 60 °C, T90—turmeric dried at 90 °C, TFD—turmeric freeze-dried, GAE—gallic acid equivalent, QE—quercetin equivalent, and CE—catechin equivalent.

**Table 2:** Antioxidant activities of control and dried samples.

Sample	DPPH	ABTS	FRAP
	IC_50_ (mg/mL or µg/mL)		µmolFe^2+^/g_dm_
CF	46.45 ± 1.82 ^c^	0.82 ± 0.05 ^a^	5.01 ± 0.14 ^a^
CRT	13.88 ± 0.50 ^a^	1.02 ± 0.03 ^b^	14.21 ± 0.88 ^c^
C60	17.81 ± 0.94 ^b^	1.38 ± 0.06 ^c^	11.28 ± 0.04 ^b^
C90	20.16 ± 0.60 ^b^	1.33 ± 0.03 ^c^	10.84 ± 0.01 ^b^
CFD	45.90 ± 1.68 ^c^	1.56 ± 0.07 ^d^	5.52 ± 0.12 ^a^
PF	26.98 ± 1.70 ^c^	1.21 ± 0.05 ^b^	12.47 ± 0.25 ^b^
PRT	13.25 ± 0.12 ^a^	0.92 ± 0.04 ^a^	16.05 ± 0.27 ^c^
P60	19.66 ± 0.27 ^b^	1.80 ± 0.02 ^d^	10.37 ± 0.11 ^a^
P90	19.97 ± 0.31 ^b^	1.72 ± 0.02 ^cd^	10.26 ± 0.30 ^a^
PFD	18.33 ± 0.16 ^b^	1.59 ± 0.08 ^c^	12.10 ± 0.20 ^b^
TF	339.33 ± 3.89 ^d^	27.84 ± 0.28 ^b^	409.67 ± 13.33 ^a^
TRT	197.97 ± 10.40 ^b^	18.62 ± 0.76 ^a^	493.41 ± 7.28 ^c^
T60	274.00 ± 15.22 ^c^	19.87 ± 0.65 ^a^	442.59 ± 4.67 ^b^
T90	263.66 ± 2.22 ^c^	25.72 ± 1.14 ^b^	428.90 ± 2.64 ^ab^
TFD	163.99 ± 4.73 ^a^	18.16 ± 0.64 ^a^	619.72 ± 6.40 ^d^

Note: Results for celery and parsley samples are expressed in mg/mL, while for turmeric samples, they are expressed in μg/mL. The results are presented as mean value ± standard deviation (*n* = 3). ^a–d^ Mean values with different letters in superscript in the same column among each plant sample are statistically different according to the Tukey’s HSD test (*p* < 0.05). CF—celery fresh, CRT—celery open-air dried at room temperature, C60—celery dried at 60 °C, C90—celery dried at 90 °C, CFD—celery freeze-dried, PF—parsley fresh, PRT—parsley open-air dried at room temperature, P60—parsley dried at 60 °C, P90—parsley dried at 90 °C, PFD—parsley freeze-dried, TF—turmeric fresh, TRT—turmeric open-air dried at room temperature, T60—turmeric dried at 60 °C, T90—turmeric dried at 90 °C, and TFD—turmeric freeze-dried.

**Table 3:** Color parameters L*, a*, and b* of control and dried samples.

Sample	L*	a*	b*
CF	57.47 ± 3.70 ^a^	−0.41 ± 0.02 ^b^	15.08 ± 0.35 ^a^
CRT	59.04 ± 1.50 ^a^	7.37 ± 0.25 ^d^	28.80 ± 1.54 ^d^
C60	67.93 ± 3.06 ^b^	1.06 ± 0.04 ^c^	21.75 ± 0.92 ^c^
C90	76.66 ± 7.09 ^c^	−0.40 ± 0.04 ^b^	18.12 ± 1.39 ^b^
CFD	89.77 ± 1.76 ^d^	−2.41 ± 0.14 ^a^	22.03 ± 1.32 ^c^
PF	63.08 ± 2.96 ^b^	−1.34 ± 0.12 ^b^	15.54 ± 1.30 ^a^
PRT	56.28 ± 2.32 ^a^	7.22 ± 0.21 ^e^	29.56 ± 2.05 ^c^
P60	63.90 ± 3.67 ^b^	0.29 ± 0.03 ^c^	21.32 ± 1.60 ^b^
P90	63.78 ± 1.61 ^b^	0.73 ± 0.06 ^d^	22.07 ± 0.75 ^b^
PFD	89.22 ± 1.64 ^c^	−2.02 ± 0.46 ^a^	20.33 ± 3.76 ^b^
TF	58.41 ± 2.92 ^b^	25.60 ± 2.23 ^b^	60.82 ± 4.33 ^c^
TRT	53.14 ± 2.20 ^a^	20.58 ± 1.60 ^a^	47.79 ± 3.42 ^a^
T60	53.25 ± 3.43 ^a^	21.81 ± 1.04 ^a^	56.77 ± 3.79 ^b^
T90	53.25 ± 2.89 ^a^	21.82 ± 1.73 ^a^	54.48 ± 1.30 ^b^
TFD	62.75 ± 1.88 ^c^	27.49 ± 1.33 ^c^	65.69 ± 2.14 ^d^

Note: The results are presented as mean value ± standard deviation (*n* ≥ 10). ^a–e^ Mean values with different letters in superscript in the same column among each plant sample are statistically different according to the Tukey’s HSD test (*p* < 0.05). CF—celery fresh, CRT—celery open-air dried at room temperature, C60—celery dried at 60 °C, C90—celery dried at 90 °C, CFD—celery freeze-dried, PF—parsley fresh, PRT—parsley open-air dried at room temperature, P60—parsley dried at 60 °C, P90—parsley dried at 90 °C, PFD—parsley freeze-dried, TF—turmeric fresh, TRT—turmeric open-air dried at room temperature, T60—turmeric dried at 60 °C, T90—turmeric dried at 90 °C, and TFD—turmeric freeze-dried.

**Table 4:** Correlation coefficient analysis of phenolic compounds and antioxidant activity in control and dried celery, parsley, and turmeric samples.

Sample	Tfl	Tflol	Tfl3ol	IC_50_^ABTS^	IC_50_^DPPH^	FRAP
TPh	0.994 **	0.984 **	0.982 **	−0.910 **	−0.759 **	0.999 **
Tfl		0.989 **	0.987 **	−0.889 **	−0.744 **	0.996 **
Tflol			0.963 **	−0.875 **	−0.731 *	0.991 **
Tfl3ol				−0.894 **	−0.744 **	0.982 **
IC_50_^ABTS^					0.676*	−0.904 **
IC_50_^DPPH^						−0.761 **

Note: * significant at *p* ≤ 0.01, ** significant at *p* ≤ 0.001.

**Table 5:** Model adequacy tests and residual analysis of the developed ANN model.

	χ^2^	RMSE	MBE	MPE	SSE	AARD	r^2^	Skew	Kurt	Mean	StDev	Var
TPh	0.702	0.810	0.227	13.937	9.834	26.132	0.998	−0.399	6.248	0.227	0.804	0.647
Tfl	21.001	4.427	−1.548	43.590	294.019	81.732	0.997	−2.746	7.906	−1.548	4.293	18.433
Tflol	92.861	9.310	4.381	34.256	1300.053	64.231	0.991	2.135	3.848	4.381	8.503	72.298
Tfl3ol	10.182	3.083	−1.322	47.921	142.546	89.852	0.958	−1.867	2.124	−1.322	2.883	8.310
IC_50_^ABTS^	0.045	0.205	0.074	11.251	0.632	21.095	0.920	3.365	11.862	0.074	0.198	0.039
IC_50_^DPPH^	447.391	20.434	−14.779	94.546	6263.477	177.273	0.591	0.017	−2.142	−14.779	14.607	213.364
FRAP	57.298	7.313	2.717	9.474	802.167	17.763	0.999	2.608	7.236	2.717	7.027	49.385

**Table 6:** Phenolic compound contents in selected samples of celery, parsley, and turmeric, identified and quantified by HPLC method.

Phenolic Compound	C90	PFD	TFD
		µg/100 g_dm_	
*p*-hydroxybenzoic acid	35.50 ± 0.08	8.92 ± 0.04	3.16 ± 0.02
Gallic acid	36.43 ± 0.04	4.19 ± 0.07	2.42 ± 0.02
Protocatechuic acid	1.67 ± 0.06	22.17 ± 0.07	4.32 ± 0.07
Catechin	n.d.	5.09 ± 0.06	4.92 ± 0.04
Epicatechin	n.d.	6.06 ± 0.12	4.12 ± 0.03
Caffeic acid	n.d.	2.87 ± 0.01	n.d.
Cinnamic acid	2.43 ± 0.01	4.47 ± 0.02	1.58 ± 0.02
Ferulic acid	2.12 ± 0.01	34.37 ± 0.03	1.45 ± 0.04
Apigenin	0.42 ± 0.02	4.19 ± 0.03	0.79 ± 0.01
Coumaric acid	4.15 ± 0.02	n.d.	2.94 ± 0.02
Chlorogenic acid	n.d.	n.d.	0.50 ± 0.01
Vanillic acid	n.d.	n.d.	2.26 ± 0.02
Quercetin	n.d.	n.d.	2.05 ± 0.10
Total amount	82.72	92.33	30.52

Note: The results are presented as mean value ± standard deviation (*n* = 3). C90—celery dried at 90 °C, PFD—parsley freeze-dried, TFD—turmeric freeze-dried, and n.d.—not detected.

**Table 7:** Antihyperglycemic activities of selected samples of celery, parsley, and turmeric.

Sample (Concentration)	Antihyperglycemic Activity (%)
C90 (100 mg/mL)	84.44 ± 0.62
PFD (40 mg/mL)	12.13 ± 0.00
TFD (40 mg/mL)	57.03 ± 0.15

Note: The results are presented as mean value ± standard deviation (*n* = 3). C90—celery dried at 90 °C, PFD—parsley freeze-dried, and TFD—turmeric freeze-dried.

**Table 8:** Antibacterial activities of extracts of selected samples of celery, parsley, and turmeric.

Sample	Zone of Inhibition (mm)	
	*Escherichia coli* ATCC 25922	*Staphylococcus aureus* ATCC 25923
C90	n.d.	n.d.
PFD	n.d.	n.d.
TFD	n.d.	8.67 ± 0.58
Positive control	34.87 ± 0.35	37.30 ± 0.75
Negative control (water)	n.d.	n.d.
Negative control (40% ethanol)	n.d.	n.d.

Note: The results are presented as mean value ± standard deviation (*n* = 3). C90—celery dried at 90 °C, PFD—parsley freeze-dried, TFD—turmeric freeze-dried, and n.d.—not detected zone of inhibition.

## Data Availability

The original contributions presented in the study are included in the article, further inquiries can be directed to the corresponding author.
